# Supplementary figures and images for: SecSel, a new software tool for conservation prioritization that is applicable to ordinal-scale data for multiple biodiversity features
Source: PLoS One. 2021 Jul 23;16(7):e0247737. doi: 10.1371/journal.pone.0247737 (PMC8301630; doi:10.1371/journal.pone.0247737)

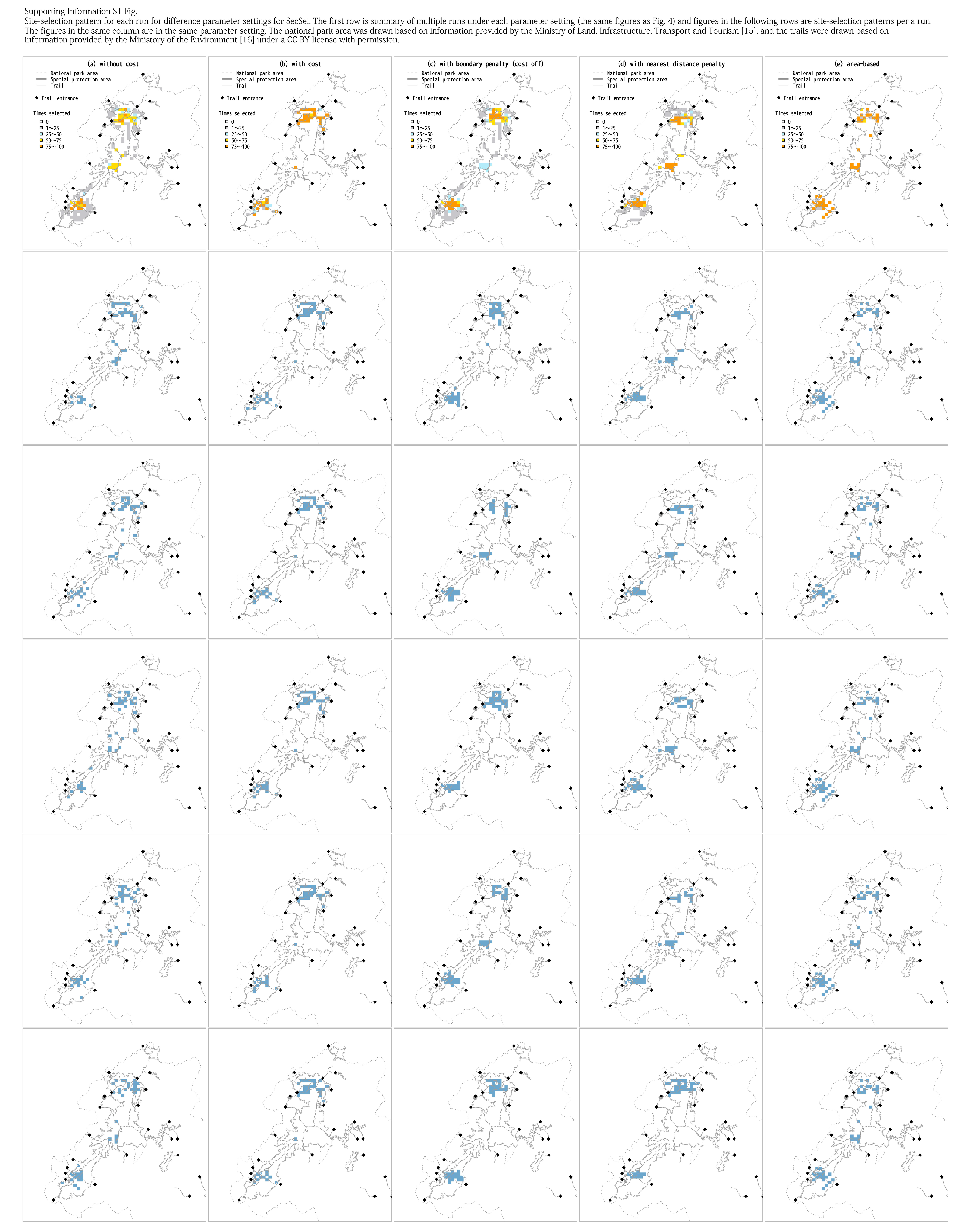

Supplement: S1 Fig — (TIF) [file pone.0247737.s001.tif]
